# Inflammation Alters the Secretome and Immunomodulatory Properties of Human Skin-Derived Precursor Cells

**DOI:** 10.3390/cells9040914

**Published:** 2020-04-08

**Authors:** Joery De Kock, Robim Marcelino Rodrigues, Steven Branson, Lieven Verhoye, Haaike Colemonts-Vroninks, Matthias Rombaut, Joost Boeckmans, Jessie Neuckermans, Sien Lequeue, Karolien Buyl, Makram Merimi, Douaa Moussa Agha, Veerle De Boe, Laurence Lagneaux, Philip Meuleman, Tamara Vanhaecke, Mehdi Najar

**Affiliations:** 1 Department of In Vitro Toxicology and Dermato-Cosmetology, Faculty of Medicine and Pharmacy, Vrije Universiteit Brussel, 1090 Brussels, Belgium; 2 Department of Clinical Chemistry, Microbiology and Immunology, Faculty of Medicine and Health Sciences, Universiteit Gent, 9000 Ghent, Belgium; 3 Laboratory of Experimental Hematology, Jules Bordet Institute, Université Libre de Bruxelles, 1000 Brussels, Belgium; 4 Genetics and Immune Cell Therapy Unit, Faculty of Sciences, University Mohammed Premier, 60000 Oujda, Morocco; 5 Department of Urology, Universitair Ziekenhuis Brussel, Vrije Universiteit Brussel, 1090 Brussels, Belgium; 6 Laboratory of Clinical Cell Therapy, Jules Bordet Institute, Université Libre de Bruxelles, 1070 Brussels, Belgium; 7 Osteoarthritis Research Unit, Department of Medicine, University of Montreal Hospital Research Center (CRCHUM), Montreal, QC H2X 3E4, Canada

**Keywords:** immunogenicity, inflammation, adult stem cells, skin stem cells, stem cell-microenvironment interactions, cytokines

## Abstract

Human skin-derived precursors (SKP) represent a group of somatic stem/precursor cells that reside in dermal skin throughout life that harbor clinical potential. SKP have a high self-renewal capacity, the ability to differentiate into multiple cell types and low immunogenicity, rendering them key candidates for allogeneic cell-based, off-the-shelf therapy. However, potential clinical application of allogeneic SKP requires that these cells retain their therapeutic properties under all circumstances and, in particular, in the presence of an inflammation state. Therefore, in this study, we investigated the impact of pro-inflammatory stimulation on the secretome and immunosuppressive properties of SKP. We demonstrated that pro-inflammatory stimulation of SKP significantly changes their expression and the secretion profile of chemo/cytokines and growth factors. Most importantly, we observed that pro-inflammatory stimulated SKP were still able to suppress the graft-versus-host response when cotransplanted with human PBMC in severe-combined immune deficient (SCID) mice, albeit to a much lesser extent than unstimulated SKP. Altogether, this study demonstrates that an inflammatory microenvironment has a significant impact on the immunological properties of SKP. These alterations need to be taken into account when developing allogeneic SKP-based therapies.

## 1. Introduction

Human skin is an easily accessible and readily available tissue that contains a broad stem cell repertoire, including hematopoietic and endothelial precursors, resident epidermal and mesenchymal stromal cells (MSC), melanocytic stem cells and so-called human skin-derived precursor cells (SKP) [1]. SKP represent a group of dermal stem/precursor cells that are obtained from primary dermal spheroids, cultivated under low attachment conditions in basic fibroblast growth factor (FGF) and epidermal growth factor (EGF) rich neurosphere medium [2]. SKP persist throughout adulthood in the human dermis [2,3] and can be isolated and expanded in large quantities from small skin biopsies of abdomen [3], breast [3], arm [4], foreskin [5], face [1] and scalp [1,6] tissue.

Historically, SKP were thought to represent a single multipotent dermal precursor cell type with multilineage differentiation capacity [2,7,8,9,10], but research by Izeta and colleagues has shown that SKP actually represent a heterogeneous group of dermal precursor cells [11]. SKP are of particular interest for regenerative medicine as they share several properties with embryonic neural crest-derived stem cells, a group of highly plastic cells with unique properties that reside within the developing embryo [2,12,13]. In particular, we and others have shown that SKP are able to generate (1) neuronal cells such as Schwann cells [7,14,15], catecholaminergic [16], dopaminergic [17] and enteric [18] neurons, (2) mesodermal cell types including adipocytes, chondrocytes, osteocytes [2] and vascular smooth muscle cells [19] and (3) endodermal progeny such as hepatic cells [5,9,20,21] and islet-like insulin-producing cells [22]. 

In the context of cell therapeutic applications of SKP, we previously reported that SKP are poorly immunogenic and are able to modulate the allogeneic immune response both in vitro and in vivo [23]. These cells therefore not only represent promising candidates for future autologous therapy, but also for allogeneic cell-based off-the-shelf applications. Their translational potential is being investigated in preclinical animal models of spinal cord injury [7,24], bone repair [8] and wound healing [25,26,27,28]. In many of these degenerative diseases, however, a pronounced inflammatory microenvironment is present [29,30,31] that might increase the immunogenicity of SKP, facilitate their rejection and thus hamper their future therapeutic application.

In this study, we investigated the impact of pro-inflammatory stimulation on the biological properties, secretome, immunogenicity and immunosuppressive capacity of SKP. Briefly, we first defined the impact of an inflammatory environment on the cell viability, size and granularity of SKP, because it has been shown for other postnatal stem cells, e.g., MSCs, that the loss of typical fibroblast-like spindle shape and differences in granularity, can lead to elevated morphological abnormality, inhomogeneity and reduced differentiation capacity [32]. In the context of cellular immunogenicity, we investigated whether inflammation induces changes in the secretion of growth factors, chemokines and cytokines by SKP and whether SKP become more immunogenic. Most importantly, we examined if pro-inflammatory stimulated SKP maintain their immunosuppressive capacity in vitro and in vivo. 

## 2. Materials and Methods

### 2.1. Isolation and Cultivation of Human SKP

SKP were isolated and subcultivated as previously described [5]. As all foreskin samples were derived from young children, informed consent was obtained from their legal guardians. All experiments were approved by the Ethical Commission of the UZ Brussels and were performed in accordance with their guidelines and regulations. The median age of the donors was 3 years (male, range 1–12 years old) and samples from a total of 9 donors were used throughout the experiments. Approximately two weeks after isolation, SKP spheroids are broken down to single cells using 0.2 mg/mL Liberase DH (Sigma-Aldrich, Overijse, Belgium) for 12 min at 37 °C. They are subsequently cultivated for 24 h in SKP growth medium supplemented with 5% (*v*/*v*) fetal bovine serum (FBS; Hyclone, Perbio, Aalst, Belgium) to promote adherence of the cells to the plastic. The next day, the attached SKP are washed and further cultivated in SKP growth medium without FBS until further use. A cell density of 2 × 10^4^ cells/cm^2^ is applied. SKP between passages 2 and 4 are used for further experiments.

### 2.2. Pro-Inflammatory Stimulation

The impact of an inflammatory environment on SKP is evaluated as previously described [33]. Briefly, SKP are stimulated for 18 h using a cocktail of pro-inflammatory cytokines: 25 ng/mL interleukin (IL)-1β (Peprotech, Rocky Hill, USA), 1 × 10^3^ U/mL interferon (IFN)-γ, 50 ng/mL tumor necrosis factor alpha (TNFα), and 3 × 10^3^ U/mL IFN-α (all from Prospec Inc, Rehovot, Israel). 

### 2.3. Acquisition and Activation of Immune Cells for In Vitro Assays

Human peripheral blood (PB) samples were collected from healthy donors after informed consent was obtained. All blood collections were approved by the Ethical Commission of the Jules Bordet Institute and performed in accordance with their guidelines and regulations. Peripheral blood mononuclear cells (PBMC) are isolated by Ficoll-Hypaque (Sigma-Aldrich, Overijse, Belgium) gradient centrifugation of PB. Total CD3^+^ T-lymphocytes are purified by positive selection using magnetic-activated cell sorting (MACS) technology (Miltenyi Biotec GmbH, Leiden, The Netherlands) according to the manufacturer’s instructions. The purity of the selected cells is always above 95% as determined by flow cytometry. Subsequently, purified CD3^+^ T-cells are activated (ST; stimulated T-cells) or not (NST; not stimulated T-cells) with 5 µg/mL of phytohemagglutinin (PHA; Remel, Thermo-Scientific, Merelbeke, Belgium) and 20 U/mL of IL-2 (Biotest AG, Germany).

### 2.4. Morphology

Morphological changes are assessed by phase contrast microscopy (100×) using an inverted microscope (Leica, Machelen, Belgium).

### 2.5. Viability Assay

SKP viability is determined by using the BD Via-Probe^TM^ viability staining solution (7-amino-actinomycin D; 7-AAD). Briefly, SKP are harvested using Tryple Select (Thermo Scientific, Merelbeke, Belgium) and stained with 0.25 µg of the nucleic acid dye 7-AAD during 10 min at room temperature. Stained SKP are immediately analyzed with the MACSQuant flow cytometer (Miltenyi Biotec GmbH, Leiden, The Netherlands). 

### 2.6. Cell Size and Granularity

The cell size (µm) of SKP is defined by flow cytometry-based comparison to standard microbeads (Miltenyi Biotec GmbH, Leiden, The Netherlands) whereas their granularity is determined by evaluating the median fluorescence intensity (MFI) of side scatter (SSC).

### 2.7. Microarray Data Analysis

Total RNA is extracted using the TriPure Isolation Reagent^TM^ (Roche Applied Science, Overijse, Belgium) and quantified at 260 nm using a Nanodrop^TM^ spectrophotometer (Thermo Scientific, Merelbeke, Belgium). The microarrays are performed as previously described using Affymetrix Human Genome U133 plus 2.0 arrays (Affymetrix, Thermo Scientific, Merelbeke, Belgium) [10]. Background correction, summarization (median polish) and normalization (quantile) are done with Robust Multiarray Analysis [34]. The data discussed in this publication have been deposited in the NCBI Gene Expression Omnibus and are accessible through GEO Series accession number GSE48757. Heatmaps and volcano plots are generated using the Transcriptome Analysis Console (TAC) version 4.0 (Applied Biosystems, Thermo Scientific, Merelbeke, Belgium).

### 2.8. Immunogenicity Assay

Prior to the assay, 1 × 10^5^ allogeneic SKP are plated for pre-adherence and treated (SKP+INFL) or not (SKP) with the inflammatory cytokine cocktail for 18 h, as described above. T-cells are activated with 5 µg/mL of PHA (Remel, Thermo Scientific, Merelbeke, Belgium) and 20 U/mL of IL-2 (Biotest AG, Dreieich, Germany) as described above, and then added to SKP cultures for 5 days of coculture. T-cell proliferation is assessed by carboxyfluorescein diacetate N-succinimidyl ester (CFDA-SE) labeling using the CellTrace^TM^ CFSE Cell proliferation kit (Thermo Scientific, Merelbeke, Belgium) following the protocol described by the manufacturer. Briefly, 2.5 × 10^5^ CD3^+^ T-lymphocytes are stained with 10 µM of CFDA-SE prior to coculture with SKP(+INFL). After the coculture period, CFSE-based fluorescence is analyzed by flow cytometry.

### 2.9. Secretion Assay

Human cytokine (ab133998) and growth factor (ab134002) antibody arrays (both from Abcam, Cambridge, UK) are used to detect major changes in the secretion profile of SKP upon proinflammatory stimulation. Briefly, 1 × 10^6^ SKP are stimulated or not with the pro-inflammatory cocktail, as described above, and culture medium is collected after 72 h. The antibody array assay is performed according to the manufacturer’s instructions and chemiluminescent detection is done on a ChemiDoc^TM^ MP Imaging System (Bio-rad, Temse, Belgium). Densitometry is performed to evaluate relative changes in the secretion profile of SKP stimulated or not with the pro-inflammatory cocktail using Bio-rad’s Image Lab v.5.2.1 software. 

Relative secretion levels are calculated as follows: summed signal intensities for each marker of interest are used. Background correction is done by subtracting the average summed signal intensities of the negative control spots. Data normalization across arrays is accomplished by defining one array as a “reference” to which the other arrays are normalized using the average summed signal intensities of the positive control spots. Next, for each marker of interest, the average summed signal intensities of the respective medium controls, either with or without pro-inflammatory cocktail, are subtracted from the samples. Finally, the obtained secretion levels for SKP with pro-inflammatory stimulation are calculated as fold change versus control SKP.

### 2.10. Cotransplantation of PBMC and SKP in SCID Mice

The local Ethical Committee for Laboratory Animal Experiments of the University of Ghent approved all described mice experiments. Briefly, human PBMC are isolated from a buffy coat (Blood Transfusion Centre - Ghent) by isopycnic density gradient centrifugation. Hu-PBL-SCID mice are produced essentially as described before [35]. Briefly, one day before transplantation, SCID mice (Prkdc^scid^/Prkdc^scid^) receive total body irradiation (300 rad) and are injected intraperitoneally with 1 mg of TM-β1, a rat monoclonal antibody that targets the β-chain of the murine IL-2 receptor. It was previously shown that TM-β1 pretreatment efficiently depletes mouse NK cells in vivo [36]. In the spleen of the SCID mice, either 5 × 10^6^ PBMC, 1 × 10^6^ SKP, 1 × 10^6^ SKP+INFL or a mixture of 5 × 10^6^ PBMC and 1 × 10^6^ SKP or SKP + INFL (5:1 cell ratio) is injected. One, two, three and four weeks after transplantation, mice are weighed and mouse EDTA plasma is collected. The concentration of human IgG is measured with a human IgG ELISA Quantitation Set (Bethyl Laboratories, Montgomery, AL, USA) according to the protocol provided by the manufacturer.

### 2.11. Statistical Analysis

The results are expressed as the mean ± standard error of the mean (SEM). For statistical comparison of more than two groups in the immunogenicity assay, a one-way ANOVA with Bonferroni’s correction is performed. For statistical comparison of the untreated versus the pro-inflammatory condition, the unpaired nonparametric two-tailed Mann-Whitney U test is performed. A *p*-value less than 0.05 is considered statistically significant (Prism v5.0d, Graph-Pad Software, La Jolla, CA, USA).

## 3. Results

### 3.1. Inflammation Alters the Cytokine and Growth Factor Secretion Profile of SKP

**Figure 1:**
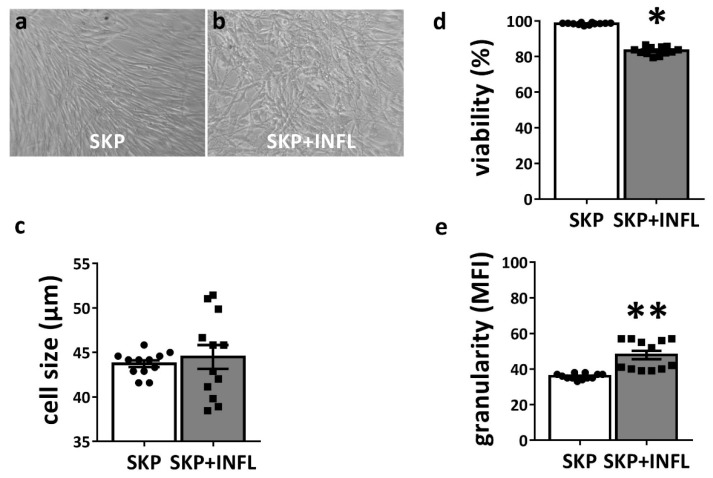
Impact of inflammation on biological properties of SKP. SKP preserve (**a**,**b**) their fibroblast-like morphology, but show (**c**) increased variation in cell size upon pro-inflammatory stimulation. The (**d**) viability and (**e**) granularity of SKP are determined under normal and inflammatory conditions. The values are expressed as mean ± SEM of four different SKP donors. * Significantly decreased versus SKP (*p*-value < 0.0001); ** Significantly increased versus SKP (*p*-value < 0.0001).

To assess the impact of an inflammatory environment, SKP are stimulated with a cocktail of pro-inflammatory cytokines (SKP + INFL) for 18h. Upon pro-inflammatory stimulation, SKP preserve their fibroblast-like morphology and mean cell size (Figure 1a–c). However, a significant decrease in cell viability (approximately 15%) could be observed for SKP + INFL (83.28 ± 0.66%) compared to SKP (98.56 ± 0.17%) (Figure 1d). A significant increase in cellular granularity and more variation in cell size was also observed, indicating increased population heterogeneity (Figure 1c,e). 

**Figure 2:**
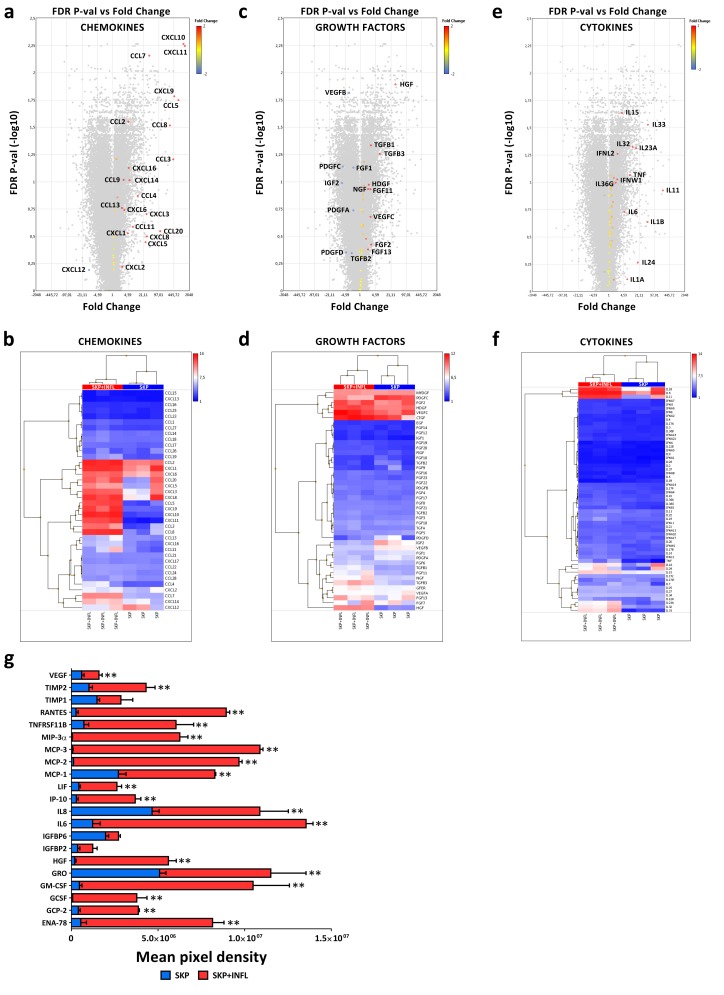
Inflammation alters the secretome of SKP and leads to drastically increased HGF secretion levels. Volcano plots and heatmaps representing (**a**,**b**) chemokine, (**c**,**d**) growth factor and (**e**,**f**) cytokine mRNA expression changes in SKP + INFL versus SKP. (**g**) Superimposed graph showing the protein secretion levels in SKP (blue bar) and SKP + INFL (blue + red bar). The values are expressed as mean ± SEM and originate from at least 3 different SKP donors. ** Significantly increased secretion versus SKP (*p*-value < 0.05).

Whole transcriptome and secretome analyses reveal that pro-inflammatory stimulation significantly alters the expression of chemokines, cytokines and growth factors in SKP (Figure 2a–g; Table 1). More specifically, gene expression microarray analyses show that the mRNA levels of the chemokine ligands *CCL2* (4.7-fold), *CCL5* (676.8-fold), *CCL7* (37.6-fold), *CCL8* (289.3-fold), *CXCL9* (459.5-fold), *CXCL10* (1135.8-fold) and *CXCL11* (1335.4-fold) as well as of the cytokines interleukin 15 (*IL15*; 3.7-fold), *IL23A* (15.4-fold), *IL32* (10.9-fold) and *IL33* (51.6-fold) are significantly increased in SKP upon pro-inflammatory stimulation (FDR *p*-value < 0.05; Figure 2a,b,e,f). Messenger RNA levels of the trophic factors hepatocyte growth factor (*HGF*) (32.9-fold), transforming growth factor B1 (*TGFB1*) (2.8-fold) and vascular endothelial growth factor B (*VEGFB*) (-3.0-fold) are also found to be significantly modulated (FDR *p*-value < 0.05; Figure 2c,d).

Furthermore, secretome antibody array analyses show that inflammation significantly increases the secretion of the chemokine ligands CCL2 (3.1-fold; MCP-1), CCL5 (34.2-fold; RANTES), CCL7 (159.6-fold; MCP-3), CCL8 (85.2-fold; MCP-2), CCL20 (147.5-fold; MIP-3a), CXCL1 (2.3-fold; GRO), CXCL5 (15.2-fold; ENA-78), CXCL6 (9.8-fold; GCP2) and CXCL10 (13.2-fold; IP-10). Constitutively, SKP secrete the chemokine ligands CCL2 (MCP-1), CCL5 (RANTES, CXCL1 (GRO) and CXCL10 (IP-10) as well as HGF, insulin growth factor binding protein (IGFBP) 2 and 6, IL8, leukemia inhibitory factor (LIF), tissue inhibitor of metalloproteinases (TIMP) 1 and 2, and VEGF (Table 1; Figure 2g; Supplementary Figure S1).

**Figure 3:**
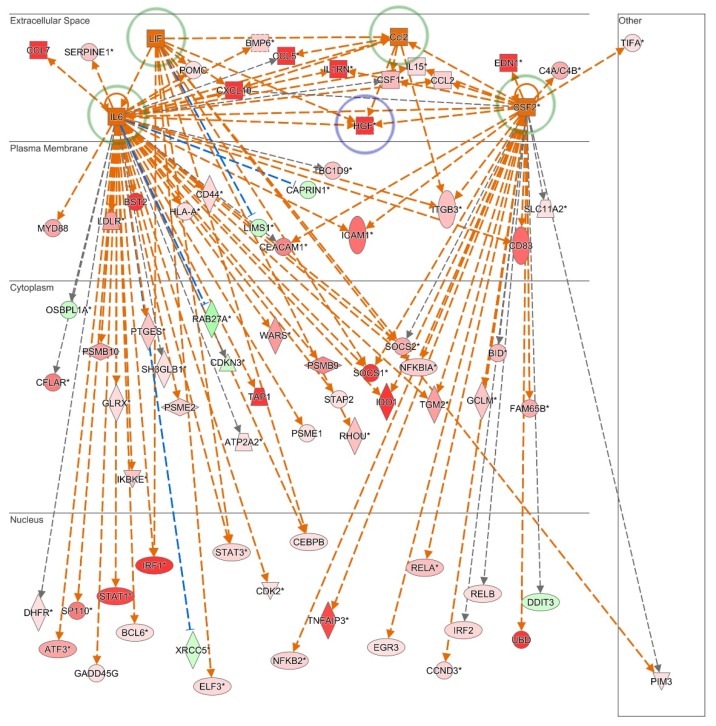
Upstream regulator interaction mapping. Mechanistic network of the upstream regulators *CCL2*, *CSF2*, *IL6* and *LIF* (green circle) that are predicted to be activated in SKP in a pro-inflammatory environment. Their combined activation is predicted to significantly contribute to the strong increase in HGF (blue circle) secretion by SKP in the presence of inflammation. Legend: red represents increased and green decreased gene expression upon pro-inflammatory stimulation. Figure produced using Ingenuity Pathway Analysis Software.

The secretion of the colony stimulating factors (CSF) 2 (22.9-fold; GM-CSF) and CSF3 (54.7-fold; GCSF), HGF (29.3-fold), IGFBP2 (3.5-fold), IL6 (11.1-fold) and 8 (2.3-fold), LIF (6.2-fold), TIMP2 (4.3-fold), tumor necrosis factor receptor superfamily (TNFRSF; 8.4-fold) 11B (also known as osteoprotegerin) and VEGF (2.7-fold) are also significantly up-regulated in SKP upon pro-inflammatory stimulation (Table 1; Figure 2a–g; Supplementary Figure S1). Moreover, in-depth upstream regulator analyses using Ingenuity Pathway Analysis software predict *CCL2* (activation z-score = 2.190), *CSF2* (activation z-score = 2.285), *IL6* (activation z-score = 5.173) and *LIF* (activation z-score = 2.492) to be activated regulators in SKP upon pro-inflammatory stimulation. Furthermore, the strong increase in HGF secretion by SKP in the presence of inflammation is evidently a direct consequence of their combined activation, as shown by pathway mapping of the respective active upstream regulators (Figure 3).

### 3.2. SKP Remain Immunosuppressive Upon In Vitro Pro-inflammatory Stimulation

**Figure 4:**
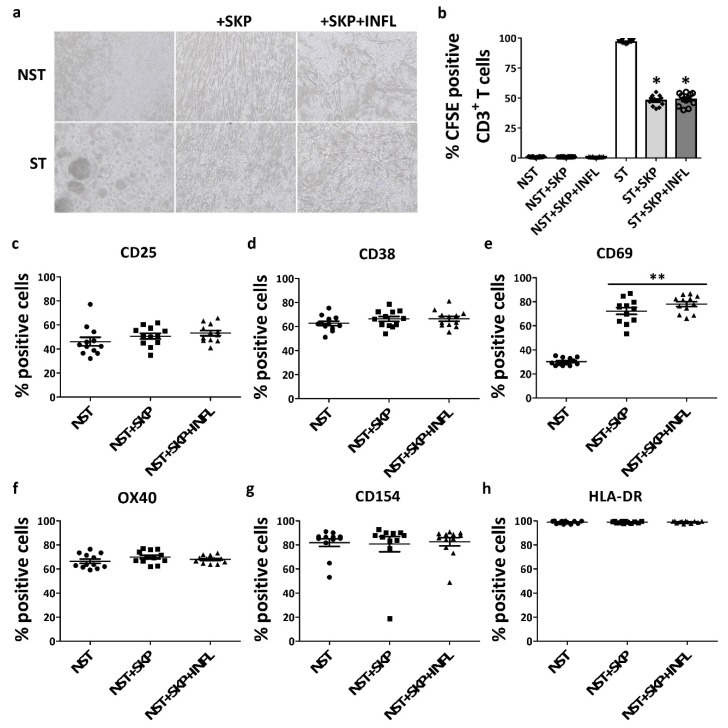
Inflammation does not alter the immunogenicity and immunosuppressive capacity of SKP towards T-cells. (**a**) Micrographs (100 X) and (**b**) flow cytometric quantification of CSFE-positive CD3^+^ T-cells in cocultures of CD3^+^ not-stimulated (NST) or stimulated (ST) T-cells with SKP with and without pro-inflammatory induction. (**c**–**h**) Flow cytometric analyses of the expression of immune regulatory molecules by NST in the presence or absence of SKP with and without pro-inflammatory induction. The values are expressed as mean ± SEM and originate from at least four different SKP donors and four different T-cell donors. * Significantly decreased percentage versus ST (*p*-value < 0.05); ** Significantly increased percentage versus NST (*p*-value < 0.05).

We observed that SKP maintain their ability to suppress T-cell proliferation in vitro after pro-inflammatory stimulation, as indicated by the absence of expanded T-cell colonies in coculture conditions (Figure 4a,b). Moreover, a significant (approximately 50%) inhibition of T-cell proliferation was achieved in both the control and inflammatory condition (Figure 4a,b). Pro-inflammatory-stimulated SKP do not initiate an allogeneic lymphocyte proliferative response, as no significant proliferation of not-stimulated CD3^+^ T-cells (NST) was observed in the presence of SKP + INFL (Figure 4a,b). 

Furthermore, NST constitutively express the immune regulatory proteins CD25 (46.22 ± 3.55%), CD38 (62.68 ± 1.84%), CD69 (30.19 ± 0.89%), OX40 (66.44 ± 1.73%; also known as CD134 and TNFRSF4), CD154 (81.88 ± 3.24%; also known as CD40L) and HLA-DR (98.78 ± 0.32%) (Figure 4c–h). Upon coculture of NST with SKP, without (72.32 ± 2.88%) and with (78.03 ± 2.06%) inflammatory stimulation, the expression of CD69 is significantly increased (Figure 4e). However, no significant changes are found in the expression of CD25, CD38, OX40, CD154 and HLA-DR by NST (Figure 4c–h).

### 3.3. Inflammation Alters the Immunosuppressive Properties of SKP In Vivo

**Figure 5:**
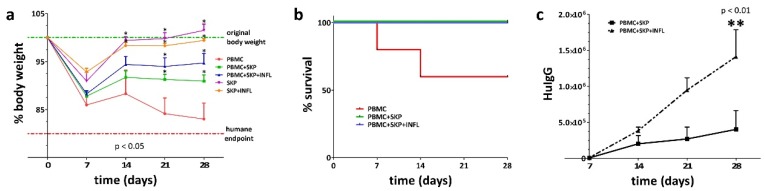
Pro-inflammatory stimulation of SKP alters, to some extent, their immune suppressive properties. (**a**,**b**) Unstimulated and pro-inflammatory stimulated SKP both suppress the graft-versus-host response of cotransplanted human PBMC in SCID mice. However, (**c**) cotransplantation of PBMC with pro-inflammatory stimulated SKP results in significantly higher human IgG blood levels compared to cotransplantation with unstimulated SKP, suggesting a lower in vivo immune suppressive capacity of SKP+INFL. * Significantly increased versus PBMC (*p*-value < 0.05); ** Significantly increased versus PBMC+SKP (*p*-value < 0.05).

To compare the in vivo immunosuppressive capacity of SKP, in the presence or absence of inflammation, we transplanted control or pro-inflammatory stimulated SKP alone, or together with human peripheral blood mononuclear cells (PBMC) in severe combined immune deficient (SCID) mice and investigated the graft-versus-host response. We observed that transplantation of both control and pro-inflammatory stimulated SKP does not have any detrimental effects on the health of the SCID mice as indicated by the normal body weight of the mice (Figure 5a). This is opposed to the transplantation of human PBMC that induce a severe graft-versus-host response and exhibit a strong negative effect on the health of the mice, as shown by the gradual loss in bodyweight and low survival rate (Figure 5b). Cotransplantation of human PBMC with control or pro-inflammatory stimulated SKP preserves the overall health status of the mice and prevents death (Figure 5a,b). Indeed, no significant differences were observed in survival time and body weight of PBMC cotransplanted mice between SKP and SKP+INFL (Figure 5a,b). However, significantly higher levels of human IgG in the blood of the mice were found on day 28 post transplantation for the PBMC/SKP+INFL (1.41 ± 0.37 ng/mL) condition compared to PBMC/SKP (0.40 ± 0.26 ng/mL), pointing to an important impairment of the immunosuppressive capacity of SKP when stimulated by pro-inflammatory factors (Figure 5c).

## 4. Discussion 

Human skin-derived precursors are a promising postnatal stem cell population for cellular therapy, as they exhibit multilineage differentiation capacity [5,7,8,16,19,22]. Furthermore, we previously showed that SKP are poorly immunogenic and exhibit favorable immunosuppressive properties [23]. As such, SKP are considered immunologically privileged, as they do not express the complete set of molecules required to fully activate T-cells, and do not stimulate their proliferation [23]. Moreover, they are able to suppress the allogeneic activation of T-cells due to a combination of direct cell contact and the secretion of soluble immune inhibitory factors, thereby inhibiting the graft-versus-host response both in vitro and in vivo [23]. It is believed that due to these features, SKP may constitute a valuable and easily accessible cell source for off-the-shelf-based cellular therapy [23]. However, one of the major safety concerns associated with stem cell-based therapies is the risk of host rejection related to unforeseen changes in the immunogenicity of the transplanted donor cells [37]. As such, patients would require life-long immune suppressant therapy. In this context, the cellular microenvironment and inflammatory milieu play key roles in determining the phenotype and the effects of immunomodulatory cells, including MSCs, on the immune system [38,39,40]. In many degenerative diseases for which cellular therapy could be considered, a pronounced inflammatory environment is often present [29,30,31]. This might increase the immunogenicity of SKP and facilitate their rejection, thus limiting their therapeutic application. Hence, potential clinical application of allogeneic SKP requires that these cells remain poorly to nonimmunogenic under all circumstances, including in a pro-inflammatory environment. In this study, we therefore investigated the impact of inflammation on the immunogenicity and immunosuppressive properties of SKP. We found that inflammation decreases the cell viability of SKP only to a limited extent. However, it also introduced a degree of heterogeneity within the cell population, as evidenced by variations in cell size and granularity. This could prelude the hampered functioning of a subset of cells within the SKP population [32]. Consequently, in cell therapy settings using SKP, a surplus of at least 15% of cells should be considered to compensate for the cell loss and population heterogeneity due to the presence of inflammation. It might therefore be important to monitor the inflammatory status of patients at the time therapeutic cells are infused to optimize cell-based therapies [41]. 

In our study, we found that pro-inflammatory-stimulated SKP maintain their immunosuppressive properties and induce the expression of CD69 in T-cells, but do not initiate an allogeneic lymphocyte proliferative response, as is also observed for unstimulated SKP. CD69 is a critical receptor for controlling regulatory T-cell (Treg)-suppressor function and acts as a regulatory molecule by modulating transforming growth factor (TGF) β levels at the site of inflammation [42,43]. Furthermore, we found that SKP increase their secretion of chemokines and cytokines involved in leukocyte chemotaxis upon pro-inflammatory stimulation, including the secretion of CCL2 (MCP-1), CCL5 (RANTES), CCL7 (MCP-3), CCL8 (MCP-2), CCL20 (MIP-3a), CXCL1 (GRO), CXCL5 (ENA-78), CXCL6 (GCP-2), CXCL10 (IP-10), IL6 and IL8 [44,45]. These chemotaxis proteins might attract immune cells to the site of transplantation and hamper their engraftment or therapeutic functioning. In addition, SKP+INFL also significantly increase their secretion of CSF2 (GM-CSF) and CSF3 (GCSF), two cytokines involved in hematopoiesis where they control the production, differentiation and function of granulocytes and macrophages [46]. An intriguing finding was the increased protein secretion of TIMP2 and OGN (TNFRSF11B) while their gene expression was decreased. This discrepancy might be explained by the fact that we quantified the extracellular secretion of proteins and not total protein production. Protein production might be—but is not necessarily—decreased when gene expression is lower, while protein secretion is increased upon exogenous stimulation. Importantly, a highly increased secretion of HGF is observed in SKP upon pro-inflammatory induction. HGF is known to be a potent immunomodulatory factor that inhibits dendritic cell function along with differentiation of IL-10-producing T-cells, promotion of regulatory T-cell generation, a decrease in IL-17-producing T-cells and down-regulation of surface markers of T-cell activation [47,48,49]. Previously, we showed that HGF secretion is mandatory for the immunosuppressive functions of SKP towards CD3^+^ T-cells, and that by addition of HGF-neutralizing antibodies, a significantly lower T-cell inhibition could be observed [23]. It can be speculated that the increase in HGF secretion upon pro-inflammatory stimulation preserves the immunosuppressive capacity of SKP towards CD3^+^ T-cells. Using in-depth pathway analyses, we could show that this observation is linked to the combined activation of CCL2, CSF2, IL6 and LIF. For the first time, these results provide information on the secretome of SKP, and thus, also on their paracrine signaling under normal and pro-inflammatory conditions. In addition, we found that cotransplantation of pro-inflammatory stimulated SKP with human PBMC in SCID mice also suppressed the graft-versus-host response, albeit to a lesser extent than unstimulated SKP. Injection of PBMC allowed us to investigate the impact on B-cell activation and the production of human IgG as a graft-versus-host response. Indeed, although no significant differences were observed in the survival time and body weight of cotransplanted mice between human SKP and SKP+INFL, significant higher levels of human IgG in the blood of the mice were found for PBMC+SKP+INFL compared to PBMC+SKP. These data indicate that the stimulation of SKP with pro-inflammatory cytokines, present in the inflammatory environment of degenerative diseases, hampers their immunosuppressive capacity. 

Altogether, our study demonstrates that the local microenvironment greatly influences the properties and functionality of SKP, and that these changes need to be well-understood and considered when developing future SKP-based therapies.

## Figures and Tables

**Table 1:** Secretome of SKP is Altered by Inflammation.

Gene Expression						Protein Secretion		
Gene	Probeset	Fold Change	FDR *p*-Value	Protein	SKP	SKP+INFL	Fold Change	*p*-Value
*CCL2* (*)	216598_s_at	4.7	2.81 × 10^−2^	MCP-1	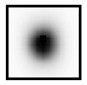	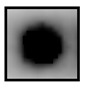	3.1	2.00 × 10^−4^
*CCL5*	1405_i_at	676.8	1.78 × 10^−2^	RANTES	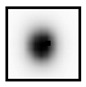	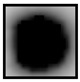	34.2	1.00 × 10^−4^
*CCL7*	208075_s_at	37.6	7.00 × 10^−3^	MCP-3	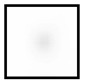	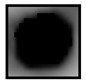	159.6	1.00 × 10^−4^
*CCL8*	214038_at	289.3	3.04 × 10^−2^	MCP-2	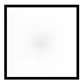	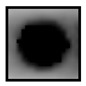	85.2	1.00 × 10^−4^
*CCL20*	205476_at	111.2	2.85 × 10^−1^	MIP-3α	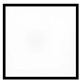	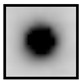	147.5	2.00 × 10^−4^
*CXCL1*	204470_at	4.4	2.97 × 10^−1^	GRO	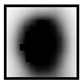	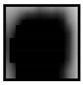	2.3	3.63 × 10^−2^
*CXCL5*	215101_s_at	25.8	3.57 × 10^−1^	ENA-78	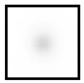	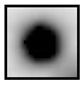	15.2	5.00 × 10^−4^
*CXCL6*	206336_at	3.2	1.82 × 10^−1^	GCP-2	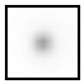	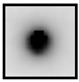	9.8	1.00 × 10^−4^
*CXCL10*	204533_at	1135.8	5.50 × 10^−3^	IP-10	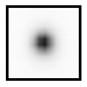	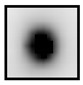	13.2	6.00 × 10^−4^
*CSF2* (*)	210229_s_at	39.1	3.98 × 10^−1^	GM-CSF	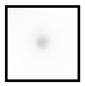	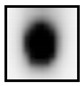	22.9	8.80 × 10^−3^
*CSF3*	207442_at	38.0	1.00 × 10^−1^	GCSF	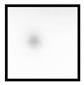	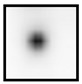	54.7	3.40 × 10^−3^
*HGF*	210997_at	32.9	1.28 × 10^−2^	HGF	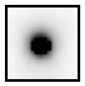	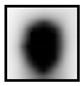	29.3	3.00 × 10^−4^
*IGFBP2*	202718_at	5.2	1.04 × 10^−1^	IGFBP2	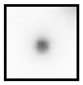	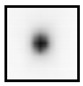	3.5	3.59 × 10^−2^
*IGFBP6*	203851_at	1.0	5.27 × 10^−1^	IGFBP6	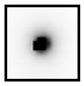	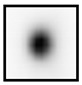	1.4	2.27 × 10^−2^
*IL6* (*)	205207_at	4.9	1.87 × 10^−1^	IL6	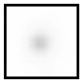	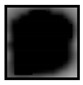	11.1	1.00 × 10^−4^
*CXCL8*	211506_s_at	29.7	3.17 × 10^−1^	IL8	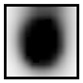	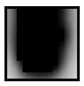	2.3	2.17 × 10^−2^
*LIF* (*)	205266_at	17.7	2.05 × 10^−1^	LIF	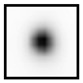	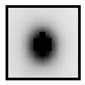	6.2	1.50 × 10^−3^
*TIMP1*	201666_at	2.1	6.81 × 10^−2^	TIMP-1	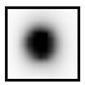	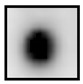	1.9	1.24 × 10^−1^
*TIMP2*	224560_at	−1.4	2.37 × 10^−1^	TIMP-2	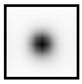	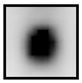	4.3	4.00 × 10^−3^
*TNFRSF11B*	204933_s_at	−5.2	9.46 × 10^−1^	OPG	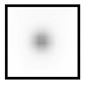	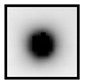	8.4	7.20 × 10^−3^
*VEGFA*	210512_s_at	−1.5	2.73 × 10^−1^	VEGF	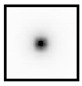	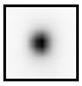	2.7	1.43 × 10^−2^
*VEGFB*	203683_s_at	−3.0	1.53 × 10^−2^					
*VEGFC*	209946_at	2.8	6.88 × 10^−2^					

* predicted to be activated as upstream regulator (activation z-score > 2).

## Supplementary Materials

The following are available online at https://www.mdpi.com/2073-4409/9/4/914/s1, Table S1: Antibodies used for flow cytometry; Figure S1: Secretome analyses of SKP upon pro-inflammatory stimulation.
