# Supplementary material for: Inflammation Alters the Secretome and Immunomodulatory Properties of Human Skin-Derived Precursor Cells
Source: Cells. 2020 Apr 8;9(4):914. doi: 10.3390/cells9040914 (PMC7226778; doi:10.3390/cells9040914)
Supplement: Supplementary file 1 [file cells-09-00914-s001.pdf]

Inflammation alters the secretome and immunomodulatory properties of human skin-derived precursor cells

Joery De Kock, Robim M. Rodrigues, Steven Branson, Lieven Verhoye, Haaike Colemonts-Vroninks, Matthias Rombaut, Joost Boeckmans, Jessie Neuckermans, Sien Lequeue, Karolien Buyl, Makram Merimi, Douaa Moussa Agha, Veerle De Boe, Laurence Lagneaux, Philip Meuleman, Tamara Vanhaecke, Mehdi Najar

Supplementary Table S1: Antibodies used for flow cytometry.

Primary antibody	Order ID	Species	Dilution	Source
anti-HLA-DR-PerCP	347402	mouse	1/20	BD
anti-CD25-FITC	F0801	mouse	1/20	Dako
anti-CD38-PE	130-098-907	mouse	1/20	MB
anti-CD69-PE	130-092-160	mouse	1/20	MB
anti-CD134-FITC (OX40)	350006	mouse	1/20	BL
anti-CD154-APC	17-1548-42	mouse	1/20	EB

Abbreviations: BD: BD Biosciences; BL, BioLegend; EB: eBioscience; MB, Miltenyi Biotec.

Supplementary Figure S1: Secretome analyses of SKP upon pro-inflammatory stimulation using antibody arrays.

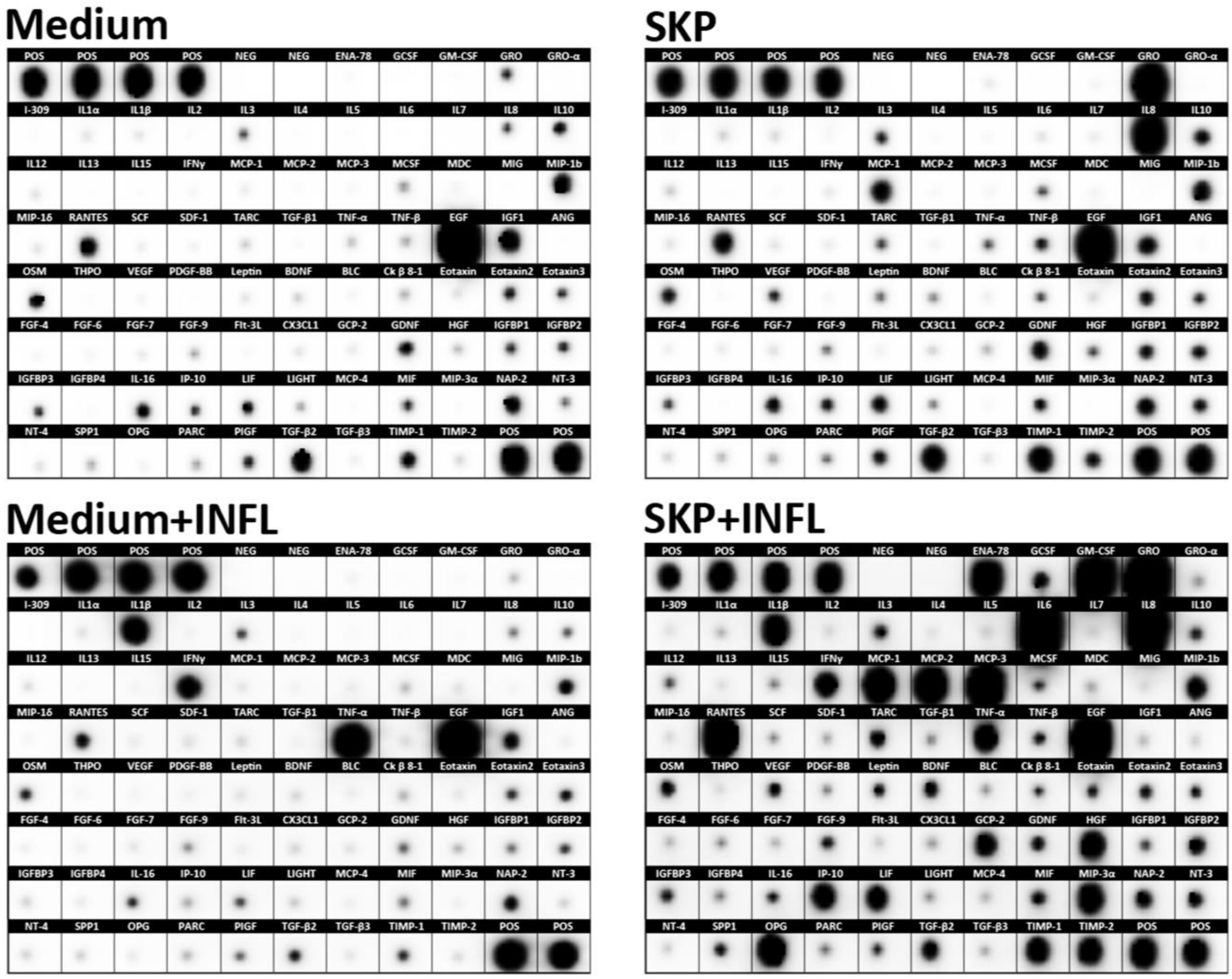

Representative membrane antibody arrays of the SKP secretome without (top) and with (bottom) pro-inflammatory stimulation and their respective medium controls.
